# The Relationship Between Aortic Arch Calcification and Recurrent Stroke in Patients With Embolic Stroke of Undetermined Source—A Case-Control Study

**DOI:** 10.3389/fneur.2022.863450

**Published:** 2022-04-25

**Authors:** Xiaofeng Cai, Yu Geng, Sheng Zhang

**Affiliations:** Center for Rehabilitation Medicine, Department of Neurology, Zhejiang Provincial People's Hospital, Affiliated People's Hospital, Hangzhou Medical College, Hangzhou, China

**Keywords:** aortic arch calcification, embolic stroke of undetermined source (ESUS), recurrent stroke, chest CT scan, AoAC grading scale

## Abstract

**Background:**

Aortic arch calcification (AoAC) is associated with plaque development and cardiovascular events. We aimed to estimate the predictive value of AoAC for stroke recurrence in patients with embolic stroke of undetermined source (ESUS).

**Methods:**

Consecutive patients with ESUS who were admitted to our center between October 2019 and October 2020 and who had a 1-year follow-up of stroke recurrence were retrospectively reviewed. According to our AoAC grading scale (AGS), AoAC was classified into four grades based on chest computed tomography (CT) findings: no visible calcification (grade 0), spotty calcification (grade 1), lamellar calcification (grade 2), and circular calcification (grade 3).

**Results:**

Of the 158 patients with ESUS (age, 62.1 ± 14.5 years; 120 men) enrolled, 24 (15.2%) had recurrent stroke within a 1-year follow-up. The Cox regression analysis showed that stroke history [hazard ratio (*HR*), 4.625; 95% confidence interval (*CI*), 1.828–11.700, *p* = 0.001] and AoAC (*HR*, 2.672; 95% *CI*, 1.129–6.319; *p* = 0.025) predicted recurrent stroke. AGS grade 1 was associated with a significantly higher risk of stroke recurrence than AGS grade 0 (*HR*, 5.033; 95% *CI*, 1.858–13.635, *p* = 0.001) and AGS grade 2 plus 3 (*HR*, 3.388; 95% *CI*, 1.124–10.206, *p* = 0.030). In patients with AoAC, receiver operating characteristic (ROC) analysis showed that AGS had a good value in predicting stroke recurrence in patients with ESUS, with an area under curve (AUC) of 0.735 (95% *CI* = 0.601–0.869, *p* = 0.005).

**Conclusions:**

Aortic arch calcification, especially spotty calcification, had a good predictive value for stroke recurrence in patients with ESUS.

## Introduction

Embolic stroke of undetermined source (ESUS) is a new clinical entity with specific diagnostic criteria, such as (1) non-lacunar stroke on neuroradiological imaging, (2) no arterial stenosis >50% or occlusion in a corresponding large artery, (3) lack of a major cardioembolic source, and (4) lack of other determined stroke causes (1). Two large randomized controlled trial studies on secondary prevention in ESUS, the NAVIGATE–ESUS trial on rivaroxaban and dabigatran, showed that the secondary prevention effect of anticoagulants was not superior to aspirin and was associated with a higher risk of bleeding in the investigated population (2, 3). These findings indicate that the etiology of ESUS is not necessarily due to an undetected cardiogenic stroke. Recent studies have shown that the stroke mechanism underlying ESUS includes low-embolic risk cardiac diseases, paradoxical brain embolisms, aortic lesions, and mild-to-moderate carotid arterial disease (4–7). Therefore, the mechanism of recurrent stroke in ESUS and its secondary prevention strategies remains controversial.

Arterial calcification has long been considered a complication of advanced atherosclerosis (8, 9). Aortic arch calcification (AoAC) is a predictor of systemic atherosclerosis, which has been shown to be associated with the occurrence of cardiovascular and cerebrovascular events, such as ischemic cerebral infarction (9, 10). In recent years, studies have reported that complex AoACs are common in patients with ESUS (10). This finding suggests that AoAC may be involved in the occurrence and recurrence of ESUS.

Previous studies based on X-rays, such as the aortic arch calcification (AAC) grading scale (8, 11), calcification in the aortic arch, age, and multiple infarction (CAM) score (11), showed that the severity of AoAC was related to the recurrence of vascular events in patients with ESUS. However, these methods have limitations in clinical applications. First, a precise evaluation of the extent of calcification seems impossible on radiography, and the relationship between the amount of calcium involvement and plaque vulnerability cannot be evaluated using X-rays (12). Second, the degree of calcification assessed by X-ray may not be consistent with the true pathological stages of calcification, and the relationship between each degree of AoAC and stroke recurrence on ESUS is not yet clear. Recently, a new pathological classification system was developed to assess the calcium burden from a healthy artery with no calcification to the advanced calcific deposits spread throughout the tunica media (13). To investigate the relationship of AoAC and its severity with stoke recurrence of ESUS, based on this pathologic classification system, we generated an AoAC grading scale (AGS) on chest computed tomography (CT), and we tested the predictive value of AGS for stroke recurrence in patients with ESUS.

## Methods

### Ethics Statement

The local ethics committee approved the study protocol. All clinical investigations were conducted in accordance with the principles of the Declaration of Helsinki.

### Patients

Between October 2019 and October 2020, we retrospectively reviewed consecutive patients who had been admitted for acute ischemic stroke within 7 days at Zhejiang Provincial People's Hospital, China. Patients were enrolled if (1) they met the ESUS diagnostic criteria adopted the criteria proposed by the ESUS International Organization (14) and (2) completed a 1-year follow-up of stroke recurrence. Patients were excluded if (1) clinical or imaging data were incomplete and (2) imaging data were not available due to motion artifacts.

### Imaging Protocol

Baseline non-contrast CT was performed using a 64-slice CT scanner (15). During hospitalization, the patients were required to undergo cranial imaging within 3–5 days after admission. All cranial MRIs in our study were performed using a 3.0T MR scanner (16). The sequences of cranial MRIs were as follows: T1WI [repetition time (TR)/echo time (TE): 160/3.05 ms], T2WI (TR/TE: 6,000/100 ms), T2-fluid-attenuated inversion recovery (FLAIR) (TR/TE: 9,000/94.0 ms), and DWI (TR/TE: 6,400/86.0 ms, *b* value 0, and 1,000 s/mm^2^). In all sequences, slice thickness and slice spacing were set as 5 and 1.5 mm, respectively. New ischemic brain lesions were defined as hyperintense lesions on postoperative brain DWI.

### Aortic Arch Calcification Grading Scale (AGS)

**Figure 1:**
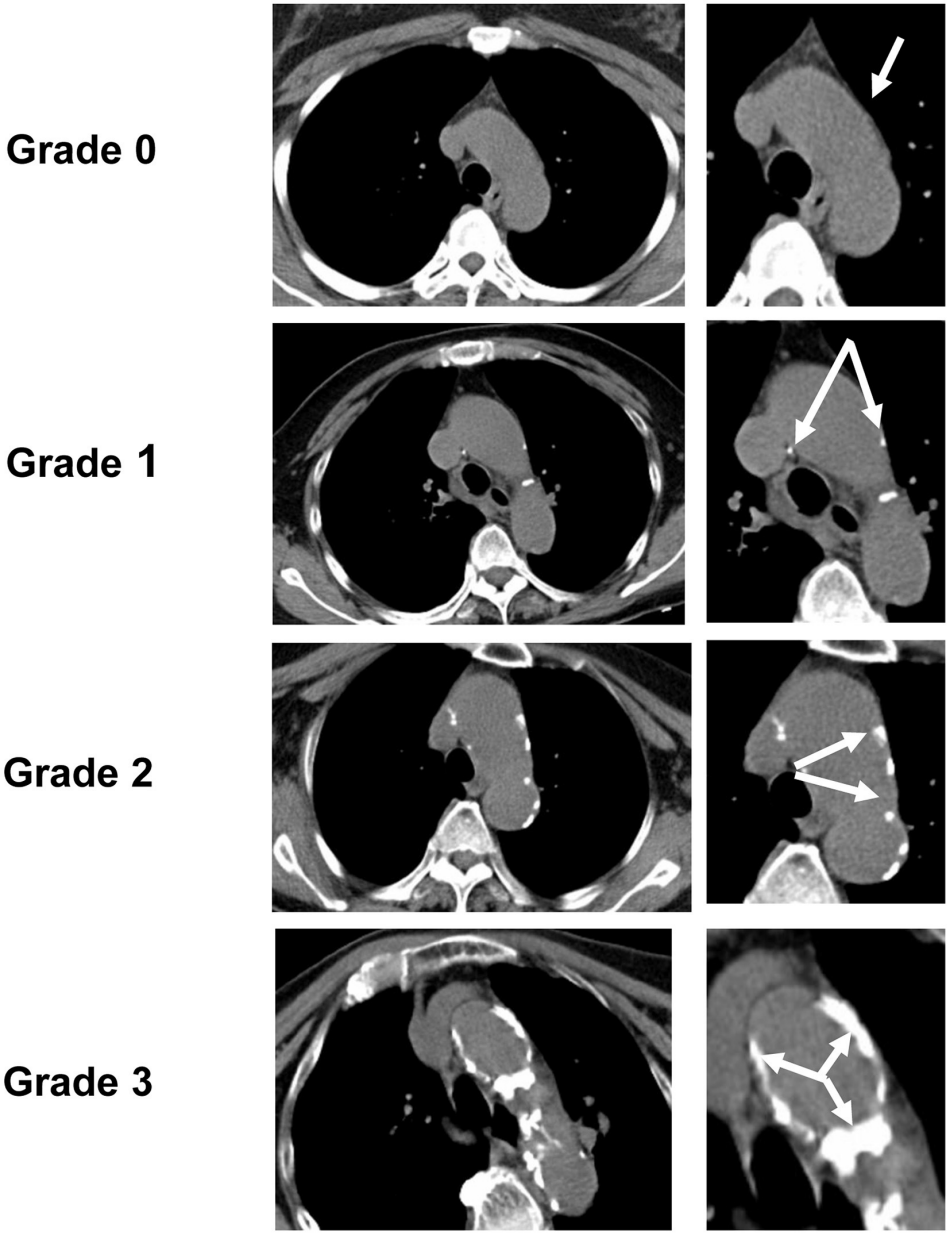
Aortic arch calcification grading scale (AGS). According to AGS, the degree of aortic arch calcification (AoAC) detected by chest CT was divided into four grades (the white arrow points to the calcification area): grade 0, no visible calcification; grade 1, spotty calcification of the aortic arch ≤ 1 mm in diameter; grade 2, lamellar calcification > 1 mm in diameter; grade 3, circular calcification.

Based on the pathological staging of calcification (13, 17), spotty calcification was defined as calcification with a diameter of ≤ 1 mm in one or more areas of the aortic intima. As CT scans are very sensitive to calcification, we applied this standard to CT and defined calcification with a diameter of no more than 1 mm as grade 1 on the AGS. Pathologically, calcification with a diameter of 1–3 mm was defined as fragment calcification, and calcification >3 mm was defined as sheet-like calcification. Therefore, on CT, we defined this type of calcification with a diameter >1 mm as grade 2 AGS. Finally, an entirely calcified artery or circular-like calcification was ascribed to an AGS grade 3. Examples of each AGS grade are shown in Figure 1.

Since this AGS is a grading of calcification severity, when multiple degrees of calcification occurred in one patient, the grade of the most serious calcification was considered as the group of that patient. For example, when patient A had both spotty and lamellar calcifications simultaneously, patient A should be ascribed to a group of lamellar calcifications.

Two raters (XFC and SZ), who jointly evaluated the AGS, were blinded to the imaging and clinical data of patients. A single trained observer (XFC) measured the AGS in all patients two times, at an interval of 1 month apart. The other observer (SZ) independently performed the same evaluation.

### Other Imaging Analysis

#### The Distribution of Infarction

New ischemic brain lesions were defined as hyperintense lesions on postoperative brain diffusion-weighted imaging. According to the distribution of infarctions, patients were divided into single territory and multiple territory infarctions.

#### Carotid Artery Ultrasound

Ipsilateral non-stenosing carotid plaque was defined using the site investigators' reports of cervical large-artery atherosclerotic plaques and infarct location. According to ultrasound echo, the characteristics of carotid plaques were defined as hypodense, hyperdense, or iso-dense.

#### Left Atrial Diameter (LAD) and Left Ventricular Ejection Fraction (LVEF)

All patients underwent transthoracic echocardiography (TTE) examination during hospitalization, and data, such as left atrial diameter (LAD), LAD/height (LAD/H), LAD/body surface area (LAD/BSA), and LVEF were recorded. LVEF estimation was based on TTE performed within 7 days after stroke. The LVEF was calculated using the Simpson biplane method (18).

### Outcome

The primary outcome was recurrent strokes. If any of the following items of the Sacco criteria (19) were satisfied, stroke recurrence could be diagnosed.

All patients were evaluated through outpatient or telephone follow-up. The clinician (XF.C., 8-year experience of in stroke management) was responsible for determining recurrent stroke.

### Statistical Analysis

Statistical analyses were performed using SPSS version 24.0. Kappa statistics were used to test inter- and intra-observer reliability for evaluating AGS. Excellent inter- and intra-observer reliabilities were observed in assessing the AGS score (κ = 0.908 and 0.867, respectively). Demographic and baseline characteristics and imaging features were reported using descriptive statistics. Numerical and nominal variables are expressed as mean standard deviation (SD) and frequency percentage, respectively. A *t*-test was used to compare the normally distributed data between groups, and the rank-sum test was used to compare the non-normally distributed data. Counting data are expressed as frequency and percentage, and chi-square analysis was used for comparison between groups. Multivariable logistic regression analysis was performed to identify risk factors for recurrent stroke in patients with ESUS. The Cox proportional hazards model was used to explore factors associated with recurrent stroke events, such as clinical characteristics and AoAC. After univariate analysis of all clinically relevant covariates, those with *p* < 0.05 were included in the multivariable Cox model. The Kaplan–Meier method and log-rank test were used to estimate the cumulative event rates of recurrent vascular events. Statistical significance was set at *p* < 0.05.

## Results

### Clinical Characteristics of Patients With ESUS

In total, 158 patients were enrolled in this study. The median age of the patients was 62 years (interquartile range [IQR]: 53–73 years) and 75.9% were men. Of the 158 patients, 98.1% (155/158) were treated with statins, 96.8% (153/158) with antiplatelet therapy, and 7.8% (12/153) with dual antiplatelet therapy. Of the 153 patients who received antiplatelet therapy, 7.8% (12/153) were switched to anticoagulant therapy because of deep venous thrombosis (*n* = 4), pulmonary embolism (*n* = 1), vertebrobasilar dolichoectasia (*n* = 1), and atrial fibrillation (*n* = 6). Atrial fibrillation was found in four patients during follow-up and in two patients during hospitalization for recurrent stroke. Univariate Cox regression analysis showed that statin, antiplatelet, and anticoagulant therapies were not associated with stroke recurrence (all *p* > 0.05). This result has been added in Table 1. A flowchart of patient screening is shown in Figure 1. During the 1-year follow-up, 24 patients (15.2%) experienced recurrent stroke.

**Table 1:** Univariate Cox regression analysis of baseline characteristics associated with stroke recurrence.

**Variables**	**HR**	**95% CI**	***P*** **value**
Age, years	1.009	0.979–1.040	0.567
Male	1.88	0.823–4.295	0.134
Risk factors			
Hypertension	0.616	0.23–1.650	0.335
Diabetes mellitus	1.482	0.588–3.733	0.404
Dyslipidaemia	1.030	0.385–2.759	0.953
Stroke history	4.739	1.877–11.965	0.001
Chronic kidney disease	21.164	0.001–39.330	0.543
Tobacco use	0.999	0.428–2.335	0.999
Alcohol abuse	0.525	0.196–1.407	0.200
Medications			
Antiplatelet therapy	0.964	0.541–1.716	0.900
Duel antiplatelet therapy^a^	1.742	0.516–5.888	0.371
Anticoagulant therapy	1.948	0.581–6.533	0.280
Statin	0.455	0.117–1.771	0.256
BMI, kg/m^2^	0.932	0.830–1.048	0.239
TCL, mmol/L	0.827	0.478–1.430	0.496
CHO, mmol/L	0.747	0.484–1.154	0.189
LDL, mmol/L	0.784	0.466–1.320	0.361
HDL, mmol/L	0.625	0.165–2.362	0.488
HbA1C, %	0.879	0.661–1.170	0.377
NIHSS score	0.981	0.882–1.090	0.721
TEE findings			
LAD, mm	1.020	0.948–1.096	0.600
LAD/H, mm/m	1.063	0.946–1.195	0.306
LAD/BSA, mm/m^2^	1.105	0.988–1.236	0.080
LVEF %	0.974	0.930–1.020	0.266
PFO	1.552	0.463–5.202	0.477
Multiple territory infarcts	0.490	0.183–1.313	0.156
Carotid plaque features			
Ipsilateral non-stenosing carotid plaque	2.320	0.962–5.596	0.061
Grading of plaque density	1.252	0.866–1.809	0.233
Diameters of carotid artery plaque, mm	0.999	0.982–1.017	0.911
AoAC	2.819	1.206–6.590	0.017
AGS			
Grade 0	–	–	–
Grade 1	6.216	2.446–15.796	<0.001
Grade 2 plus 3^*^	1.922	0.667–5.539	0.226

a *indicates the aspirin combined with clopidogrel antiplatelet therapy*.

* *As none of the patients with AGS grade 3 had recurrent stroke, patients with AGS grades 2 and 3, marked as grade 2 plus 3, were enrolled in the univariate Cox regression analysis. HR, hazard ratio; 95% CI, 95% confidence interval; AoAC, aortic arch calcification; AGS, aortic arch calcification grading scale; BMI, body mass index; NIHSS, National Institutes of Health Stroke Scale; LAD, left atrial diameter; LAD/H, left atrial diameter/height; BSA, body surface area; LEVF, left ventricular ejection fraction; PFO, patent foramen ovale. TCL, triglyceride, CHO, cholesterol, LDL, low-density lipoprotein, HDL, high-density lipoprotein, HbA1C, glycosylated hemoglobin*.

Univariate comparisons of baseline clinical characteristics are shown in Supplementary Table 1. Compared with patients without recurrent stroke, patients with recurrent stroke were more likely to have a history of stroke (patients that had a history of stroke before enrolment in this study), left atrial diameter/height (LAD/H) enlargement, and AoAC (all *p* < 0.001). Stroke history, LAD/H, and AoAC were associated with stroke recurrence in the univariate Cox regression analysis (*p* < 0.05) (Table 1). These three factors were entered into multivariate Cox regression analysis as covariates to explore the predictors of stroke recurrence. AoAC use was significantly associated with stroke recurrence (hazard ratio [*HR*], 2.672; 95% confidence interval (*CI*), 1.129–6.319; *p* = 0.025). Stroke history was also predictive of recurrent stroke (*HR*, 4.625; 95% *CI*, 1.828–11.700, *p* = 0.001).

### The Association Between AoAC and Stroke Recurrence

Among 158 patients with ESUS, 69 (43.7%) were identified as having AoAC. A comparison of the clinical characteristics of patients with and without AoAC is shown in Supplementary Table 2. In the AoAC subgroup (*n* = 69), 22 patients had spotty calcifications (grade 1), while 37 patients had lamellar calcification (grade 2) and 10 patients had circular calcification (grade 3), according to our AGS.

In patients with no AoAC on chest CT (AGS grade 0) (*n* = 89), only eight patients (9%) experienced stroke recurrence. In patients with AoAC (*n* = 69), the risk of stroke recurrence was reduced with an increase in AoAC severity. The rate of stroke recurrence was highest (45.5%) in patients with AGS grade 1, then followed by AGS grade 2 (16.2%). Of note, none (0%) of the patients with AGS grade 3 presented with stroke recurrence during the 1-year follow-up observation. Univariate comparison analysis showed that the rate of stroke recurrence was significantly higher in patients with AGS grade 1 than in those with any other AGS grade (all *p* < 0.05), while there was no significant difference in stroke recurrence among patients with AGS grades 0, 2, and 3 (χ^2^ = 2.717, *p* = 0.257).

After replacing AoAC with AGS in multivariate Cox regression analysis, AGS grade 1 (*HR*, 5.033; 95% *CI*, 1.858–13.635, *p* = 0.001) was associated with a significantly higher risk of stroke recurrence in comparison with AGS grade 0, while AGS grade 2 plus 3 (lamellar and circular calcification) showed no higher risk of stroke recurrence than AGS grade 0 (*HR*, 1.558; 95% *CI*, 0.539–4.509, *p* = 0.413).

In the AoAC subgroup (*n* = 69), multivariate Cox regression analysis showed that AGS grade 1 was associated with a significantly higher risk of stroke recurrence than AGS grade 2 plus 3 (*HR*, 3.388; 95% *CI*, 1.124–10.206, *p* = 0.030). An ROC analysis showed that AGS had a good value for predicting stroke recurrence with an AUC of 0.735 (95% *CI* = 0.601–0.869, *p* = 0.005).

**Figure 2:**
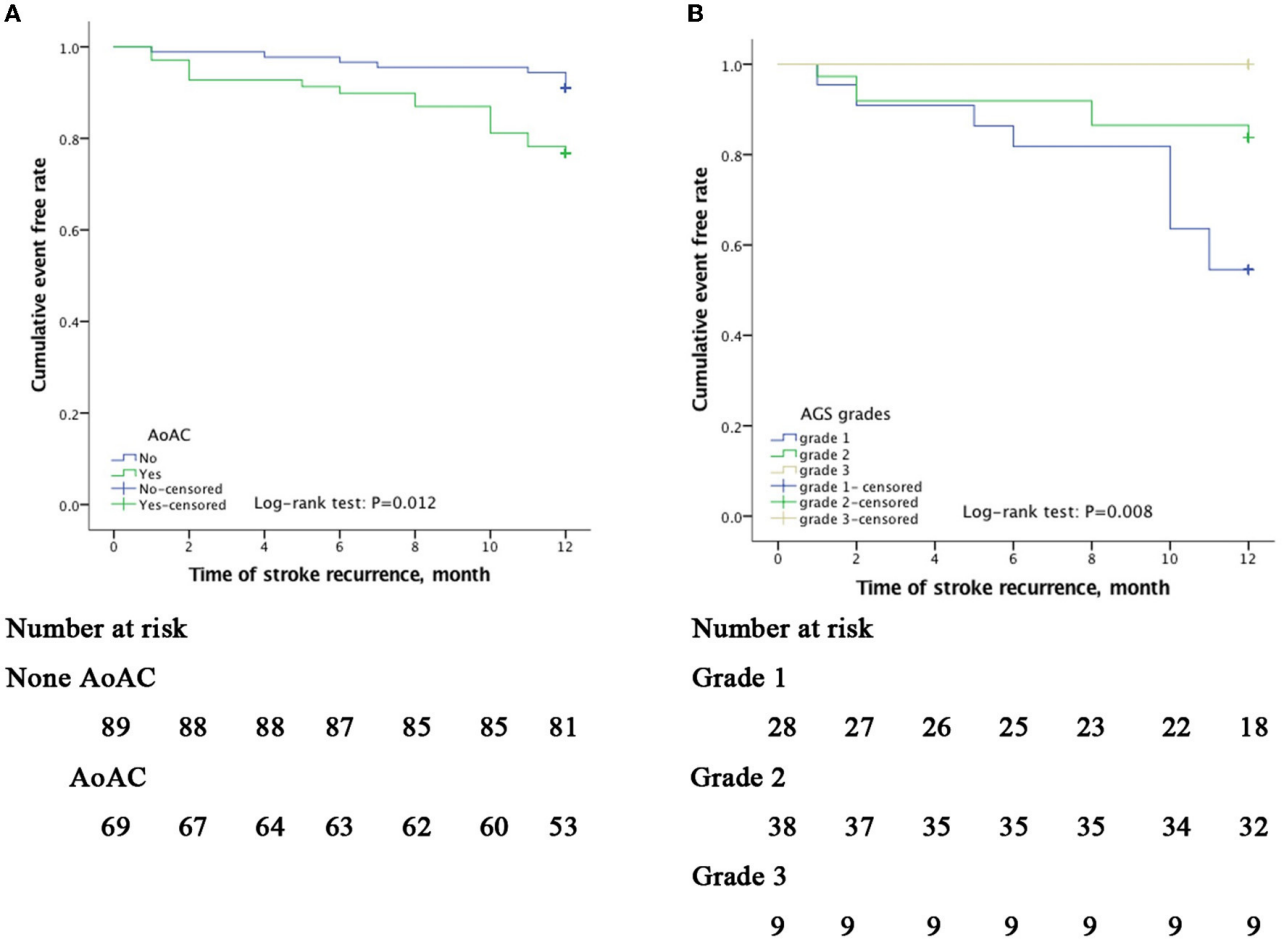
Kaplan–Meier curves of freedom from recurrent stroke events during a 12-month follow-up. The *x*-axis indicates time in month since inclusion in the study. The *y*-axis indicates the proportion of patients with recurrence-free stroke. Cumulative event-free rates were compared based on the presence of AoAC **(A)** and among AGS grade 1(spotty calcification), 2 (lamellar calcification), and 3 (circular calcification); **(B)** showing *p* = 0.012 and *p* = 0.008 on the log-rank test, respectively.

Kaplan–Meier survival curves showed that the cumulative event (stroke recurrence) free rate was significantly lower in patients with a history of stroke than in those without a history of stroke (log-rank test, *p* < 0.001) and lower in patients with AoAC than in patients without AoAC (log-rank test, *p* = 0.012; Figure 2A). In the AoAC subgroup (*n* = 69), the Kaplan–Meier survival curves showed that the cumulative event-free rate was significantly lower in patients with AGS grade 1 than in patients with AGS grade 2 and 3 (log-rank test, *p* = 0.008; Figure 2B).

## Discussion

In the present study, the 1-year stroke recurrence rate with ESUS was approximately 15%. A previous history of stroke and AoAC and its degree were demonstrated to be predictors for 1-year stroke recurrence in patients with ESUS. In contrast to the other subtypes of AoAC, spotty calcification (≤ 1 mm in diameter) was associated with a higher risk of stroke recurrence in our patients with ESUS.

First, the recurrence rate of ESUS in our study was higher than that in previous reports. In general, the prevalence of stroke recurrence in ESUS was annually approximately 2.3–13% (14, 20). We speculated that this gap was mainly caused by different study populations. It was reported that the stroke recurrence was more frequently seen in China than that in the West (21, 22). Data from the Chinese National Stroke Registry showed that the stroke recurrence rate in patients with ischemic stroke was 16% in the first year which was similar to our outcome (23, 24). According to data from the China National Stroke Screening and Prevention Project (CNSSPP), the standardized prevalence of AF among Chinese adults aged ≥40 years was 2.31%. Notably, the rate of stroke recurrence in our patients with ESUS was quite close to that reported in patients with large artery atherosclerosis (LAA) (25, 26), further supporting that large artery non-stenosing plaque might be the potential etiology of stroke and stroke recurrence in this ESUS population in China.

Second, we found that AoAC, especially spotty calcification, was a key factor in the high incidence of stroke recurrence in ESUS. Interestingly, the risk of recurrent stroke with spotty calcification is not only higher than that without calcification but also higher than that with lamellar and circular calcifications. This finding is very different from those of previous research. Some studies believe that the higher the severity of AoAC which is reflected by the AAC grade or CAM score, the higher the risk of stroke recurrence (11). However, some studies failed to find a strong correlation (27, 28). We speculated that the reasons why our results were different included (1) the study population was different and (2) the evaluation methods of calcification were different. Compared with AAC, our evaluation using CT is more objective and accurate than X-ray imaging (29). Compared with the CAM score, although our method was unable to quantify the degree of AoAC, our research method was simpler and might be easier to apply in other centers for patients with stroke. Although our results are in contrast to those of other studies, the mechanism of low-grade calcification in predicting stroke recurrence can still be logical. One important embolic source in ESUS is atherosclerotic plaque in the carotid, vertebrobasilar, and intracranial arteries, or the aortic arch collectively described as supracardiac atherosclerosis (30). A previous research has shown that spotty calcification generally occurs in the aortic intima which increases the risk of plaque rupture. Macrocalcification, such as areas of calcification and circular calcification, is more common in the tunica media of the acral arteries and often leads to stenosis (10, 11). Moreover, this suggests that plaque size has certain limitations in evaluating stroke recurrence. In the future, plaque morphology or routine plaque vulnerability assessment for AoAC may better explain the relationship between AoAC and stroke recurrence. In addition, whether intensive statin treatment can better prevent stroke recurrence in patients with this type of spotty calcification is a problem that needs to be addressed in future research.

On chest CT, AoAC, a risk marker for cardiovascular disease, becomes available in subjects with no additional radiation burden to the patient and no additional work for the radiologist. Our AGS has proven to be a valuable evaluation tool for recurrent stroke risk for patients with ESUS. For AGS grade 0, the stroke recurrence rate was much lower than that of grade 1, but was close to the incidence in the severe calcification group (grades 2–3). We propose that patients without calcification should still be evaluated for cardiogenic stroke, such as recommending screening for 24 h or longer electrocardiograms (ECGs), and patent foramen ovale (PFO). For AGS grades 2–3, more serious calcification might be associated with a relatively low risk of recurrent stroke in patients with ESUS in comparison with spotty calcification (grade 1). Further assessment of atherosclerotic plaque stability upon cardiac imaging, such as computed tomography angiography (CTA) or digital subtraction angiography (DSA), might be beneficial as a secondary prevention strategy for ESUS stroke and calcification of ESUS etiology.

Furthermore, cardiovascular calcifications were associated with cardiovascular events and death (31, 32). They can involve the coronary arteries, cardiac valves, myocardium, pericardium, and aorta artery. Different location and the level of calcifications may be potential markers in identifying patients of a high-risk phenotype for developing recurrent stroke.

This study had several limitations. First, the study design was retrospective and selection bias could not be ruled out. Second, the data from the current study were derived from a single center and the number of patients with recurrent stroke was small. Third, we did not select the Agatston score to quantify the severity of AoAC on chest CT because we considered that the AGS might be more applicable and feasible in most centers. Moreover, with the advent of coronavirus disease 2019 (COVID-19), patients with stroke must undergo routine chest CT scans at admission, which provides convenience for further stroke recurrence risk assessment with a larger sample size in the future. At last, for technical and economic reasons, implantable loop recorder monitoring for atrial fibrillation has not been carried out in our hospital. Yushan et al. found that the rate of AF detection was much higher at 12% with insertable cardiac monitor (ICM) in patients with ESUS (33). Lack of ICM might influence the detection rate of AF in our research.

In conclusion, AoAC, particularly spotty calcification on chest CT, was effective in predicting future recurrent stroke in patients with ESUS. AGS might be a valuable evaluation tool for stroke recurrence risk in ESUS, which needs to be confirmed in prospective, large-sample-sized studies in the future.

## Data Availability Statement

The original contributions presented in the study are included in the article/Supplementary Material, further inquiries can be directed to the corresponding author/s.

## Ethics Statement

The protocols of the study had been approved by the Local Ethics Committee. All clinical investigation has been conducted according to the principles expressed in the Declaration of Helsinki. The patients/participants provided their written informed consent to participate in this study.

## Author Contributions

XC and YG contributed to the conception and design of this study. XC contributed to the acquisition and analysis of the data and figures preparation. SZ contributed to the data analysis. XC and SZ contributed to drafting the manuscript. All authors contributed to the article and approved the submitted version.

## Conflict of Interest

The authors declare that the research was conducted in the absence of any commercial or financial relationships that could be construed as a potential conflict of interest.

## Publisher's Note

All claims expressed in this article are solely those of the authors and do not necessarily represent those of their affiliated organizations, or those of the publisher, the editors and the reviewers. Any product that may be evaluated in this article, or claim that may be made by its manufacturer, is not guaranteed or endorsed by the publisher.
