# Supplementary material for: The Relationship Between Aortic Arch Calcification and Recurrent Stroke in Patients With Embolic Stroke of Undetermined Source—A Case-Control Study
Source: Front Neurol. 2022 Apr 25;13:863450. doi: 10.3389/fneur.2022.863450 (PMC9084855; doi:10.3389/fneur.2022.863450)
Supplement: Supplementary file 1 [file Data_Sheet_1.docx]

**Supplementary table I.** Baseline Characteristics and Examination Findings According to Classification by Recurrent Ischemic Stroke

| Variables | All | Recurrent stroke | | Test value | *P* value |
| --- | --- | --- | --- | --- | --- |
|  |  | Present | Absent |  |  |
|  | n=158 | n=28 | n=130 |  |  |
| Age, years, median (IQR) | 62.5(53-73) | 62.5(55-72) | 62.5(52-73) | Z=0.506 | 0.613 |
| Male, n (%) | 120 (75.9) | 15(62.5) | 105（78.4） | χ^2^=2.802 | 0.095 |
| Risk factors, n (%) | | | | | |
| Hypertension | 111(70.3) | 19(79.2) | 92（68.7） | χ^2^=1.076 | 0.300 |
| Diabetes mellitus | 52(32.9) | 6(25.0) | 46（34.3） | χ^2^=0.802 | 3.720 |
| Dyslipidemia | 34(21.5) | 5(20.8) | 29（21.6） | χ^2^=0.008 | 0.930 |
| Stroke history | 12(7.6) | 6(25.0) | 6（4.5） | χ^2^=12.215 | <0.001 |
| Chronic kidney disease | 5(3.2) | 0(0) | 5（4.5） | χ^2^=0.925 | 0.338 |
| Tobacco use | 52(32.9) | 7(29.2) | 45（33.6） | χ^2^=0.002 | 0.962 |
| Alcohol abuse | 21(13.3) | 5(20.8) | 16（11.9） | χ^2^=1.397 | 0.239 |
| BMI, kg/m2，mean±SD | 24.1±3.6 | 23.4±2.6 | 24.3±3.8 | t=0.661 | 0.979 |
| TCL, mmol/L, mean±SD | 1.5±0.9 | 1.5±0.7 | 1.5±1.1 | t=0.594 | 0.110 |
| CHO, mmol/L, mean±SD | 4.2±1.1 | 4.0±1.2 | 4.3±1.0 | t=1.171 | 0.243 |
| LDL, mmol/L, mean±SD | 2.5±0.8 | 2.4±0.9 | 2.5±0.8 | t=0.768 | 0.444 |
| HDL, mmol/L, mean±SD | 1.1±0.4 | 1.0±0.3 | 1.1±0.4 | t=0.703 | 0.483 |
| HbA1C, （IQR） | 5.7(5.3-6.7) | 5.8(5.2-6.9) | 5.7(5.3-6.8) | Z=0.575 | 0.566 |
| NIHSS score, median (IQR) | 3(1-5) | 3(1-5) | 2.5(1-5.25) | Z=0.905 | 0.505 |
| Examination findings | | | | | |
| LAD, mm，mean±SD | 35.7±5.32 | 36.3±5.35 | 35.4±5.3 | t=0.728 | 0.830 |
| LAD/H, mm/m，mean±SD | 21.5±3.2 | 22.1±3.2 | 21.4±2.2 | t=0.186 | 0.013 |
| LAD/BSA，mm/m^2^，mean±SD | 20.9±3.3 | 22.0±3.2 | 20.7±3.4 | t=1.775 | 0.078 |
| LVEF %，mean±SD | 63.5±7.2 | 61.8±7.6 | 63.8±7.1 | t=0.844 | 0.691 |
| PFO，n(%) | 21(13.3) | 3(12.5) | 18(13.4) | χ^2^=0,529 | 0.468 |
| multiple territory infarcts, (n)% | 19(12.0) | 5(20.8) | 14(10.4) | χ^2^=2.075 | 0.151 |
| Carotid plaque features |  |  |  |  |  |
| Ipsilateral non-stenosing carotid plaque, n (%) | 82(51.9) | 17(70.8) | 65(48.5) | χ^2^=4.064 | 0.044 |
| Grading of plaque density, median (IQR) | 2.0(0-3) | 1.5 (1-2) | 1 (0-2) | Z=-1.207 | 0.227 |
| Diameters of carotid artery plaque, mm, mean±SD | 19.1±23.0 | 18.6±17.4 | 19.3±23.9 | t=0.126 | 0.900 |
| AoAC, n (%) | 69 | 16(66.7) | 53 (39.6) | χ^2^=10.086 | 0.001 |
| AGS, n (%) |  |  |  | χ^2^=20.120^*^ | <0.001^*^ |
| No calcification (Grade 0) | 89 | 8 (33.3) | 81 (60.4) |  |  |
| Spotty calcification (Grade 1) | 22 | 10 (41.7) | 12 (9.0) |  |  |
| Lamellar calcification (Grade 2) | 37 | 6 (25.0) | 31 (23.1) |  |  |
| Circular calcification (Grade 3) | 10 | 0 (0) | 10 (7.5) |  |  |

AoAC, aortic arch calcification; AGS, aortic arch calcification grading scale; BMI, body mass index; NIHSS, national institutes of health stroke scale; LAD, left atrial diameter; LAD/H, left atrial diameter/height; BSA, body surface area; LEVF, left ventricular ejection fraction, PFO, patent foramen ovale. TCL, triglyceride, CHO, cholesterol, LDL, low density lipoprotein, HDL, high density lipoprotein, HbA1C, glycosylated hemoglobin.

**Supplementary table II.** Univariate comparison analysis of baseline characteristics between patients with and without AoAC.

|  | no AoAC | AoAC |  |  |
| --- | --- | --- | --- | --- |
| Variables | n=89 | n=69 | Test value | *P* value |
| Age, years, median (IQR) | 56 (48-66.5) | 70 (60.5-76) | Z=-0.506 | 0.613 |
| Male, n(%) | 74 (83.1) | 46 (66.7) | χ^2^=5.778 | 0.016 |
| Risk factors, n (%) | | | | |
| Hypertension | 30 (33.7) | 17 (24.6) | χ^2^=1.530 | 0.216 |
| Diabetes mellitus | 29 (32.6) | 23 (33.3) | χ^2^=0.010 | 0.921 |
| Dyslipidemia | 20 (22.5) | 14 (20.3） | χ^2^=0.110 | 0.741 |
| Stroke history | 6 (6.7) | 6 (8.7) | χ^2^=0.211 | 0.646 |
| Chronic kidney disease | 3 (3.4) | 2 (2.9) | χ^2^=0.028 | 0.866 |
| Tobacco use | 24 (27.0) | 28 (40.6) | χ^2^=3.262 | 0.071 |
| Alcohol abuse | 13 (14.6) | 8 (11.6) | χ^2^=0.306 | 0.58 |
| BMI, kg/m2，mean±SD | 24.3±3.8 | 23.4±2.6 | t=1.107 | 0.27 |
| TCL, mmol/L, mean±SD | 1.5±1.1 | 1.5±0.7 | t=0.549 | 0.553 |
| CHO, mmol/L, mean±SD | 4.2±1.1 | 4.2±1.0 | t=0.226 | 0.791 |
| LDL, mmol/L, mean±SD | 2.6±0.8 | 2.5±0.9 | t=0.524 | 0.601 |
| HDL, mmol/L, mean±SD | 6.5±1.9 | 6.2±1.3 | t=-1.205 | 0.230 |
| HbA1C, (IQR) | 5.7(5.2-7.1) | 5.7(5.4-6.6) | Z=-0.212 | 0.832 |
| NIHSS score, median (IQR) | 3 (1-5) | 3 (1-5) | Z=-0.173 | 0.862 |
| Examination findings | | | | |
| LAD, mm，mean±SD | 35.6±5.3 | 36.3±5.4 | t=-0.578 | 0.564 |
| LAD/H, mm/m，mean±SD | 21.4±3.2 | 22.1±3.1 | t=-1.079 | 0.282 |
| LAD/BSA，mm/m^2^，mean±SD | 20.7±3.3 | 22.0±3.1 | t=-1.775 | 0.078 |
| LVEF %，mean±SD | 63.8±7.1 | 62.0±7.6 | t=1.144 | 0.254 |
| PFO，n(%) | 18 (20.2) | 10 (14.5) | χ^2^=0,876 | 0.349 |
| multiple territory infarcts, (n)% | 10 (11.2) | 9 (13.0) | χ^2^=0.120 | 0.729 |
| Carotid plaque features |  |  |  |  |
| Ipsilateral non-stenosing carotid plaque, n(%) | 37(41.6) | 45(65.2) | χ^2^=8.7 | 0.003 |
| Grading of plaque density, median (IQR) | 1 (0-2) | 2 (1-2) | Z=-1.207 | 0.227 |
| Diameters of carotid artery plaque, mm, mean±SD | 19.3±34.9 | 18.6±17.4 | t=0.126 | 0.9 |

BMI, body mass index; NIHSS, national institutes of health stroke scale; LAD, left atrial diameter; LAD/H, left atrial diameter/height; BSA, body surface area; LEVF, left ventricular ejection fraction, PFO, patent foramen ovale. TCL, triglyceride, CHO, cholesterol, LDL, low density lipoprotein, HDL, high density lipoprotein, HbA1C, glycosylated hemoglobin.
